# A novel oncolytic virus-based biomarker participates in prognosis and tumor immune infiltration of glioma

**DOI:** 10.3389/fmicb.2023.1249289

**Published:** 2023-09-22

**Authors:** Zheng Hao, Xiaofeng Yin, Rui Ding, Laizhao Chen, Chunyan Hao, Hubin Duan

**Affiliations:** ^1^Department of Neurosurgery, Second Hospital of Shanxi Medical University, Taiyuan, China; ^2^Department of Geriatrics, First Hospital of Shanxi Medical University, Taiyuan, China; ^3^Department of Neurosurgery, First Hospital of Shanxi Medical University, Taiyuan, China

**Keywords:** glioma, oncolytic virus, entrovirus, prognosis, immune infiltration

## Abstract

**Background:**

Glioma is the most common central nervous malignancy. Due to its poor survival outcomes, it is essential to identify novel individualized therapy. Oncolytic virus (OV) treatment is a key therapy regulating tumor microenvironment in malignant glioma. Herein, we aim to identify the key genes after OV infection and its role in glioma.

**Methods:**

Performing an RNA-seq analysis, the differentially expressed genes (DEGs) between EV-A71-infection and mock group were screened with GFold values. DAVID online analysis was performed to identify the functional classification. Overall survival (OS) or disease-free survival (DFS) was evaluated to analyze the relation between PTBP1 expression levels and prognosis of glioma patients. Additionally, the ssGSEA and TIMER algorithms were applied for evaluating immune cell infiltration in glioma.

**Results:**

Following EV-A71 infection in glioma cells, PTBP1, one of the downregulated DEGs, was found to be associated with multiple categories of GO and KEGG enrichment analysis. We observed elevated expression levels of PTBP1 across various tumor grades of glioma in comparison to normal brain samples. High PTBP1 expression had a notable impact on the OS of patients with low-grade glioma (LGG). Furthermore, we observed an obvious association between PTBP1 levels and immune cell infiltration in LGG. Notably, PTBP1 was regarded as an essential prognostic biomarker in immune cells of LGG.

**Conclusion:**

Our research uncovered a critical role of PTBP1 in outcomes and immune cell infiltration of glioma patients, particularly in those with LGG.

## Introduction

Glioma is a primary malignant tumor that occurs within the brain and has the highest incidence rate among tumors (Chen et al., 2017). Glioblastoma multiforme (GBM), the most aggressive form of brain cancer with a dismal prognosis, represents approximately half of all newly diagnosed gliomas (Le Rhun et al., 2019). In despite of the implementation of optimizing clinical treatment, which involve responsible surgical excision followed by chemoradiotherapy, low-grade glioma (LGG) patients have an overall survival rate of only about 60 months (Malik et al., 2021). The diffuse invasive growth of glioma cells and the blood–brain barrier are the major contributors to poor prognosis, as it not only impedes surgical tumor excision but also facilitates resistance to chemotherapy and radiotherapy (van Tellingen et al., 2015; Hu et al., 2023). Improving the extended survival rate of glioma patients remains a challenge. Due to the swift advancement of next-generation sequencing, the utilization of pan-cancer investigation has become prevalent in the detection of molecular indicators within tumors, such as aldehyde dehydrogenase (ALDH; Xia et al., 2023), Unc-51 Like Autophagy Activating Kinase 1 (ULK1; Qu et al., 2020) and N6-methyladenine-related genes (Qu et al., 2021a). The identification of novel therapeutic targets based on regulatory mechanism is crucial for enhancing patient outcomes.

Replication-competent virus is a novel oncolytic virotherapy targeting cancer cells (Russell et al., 2012). The oncolytic viruses (OVs) can trigger tumor cell apoptosis via diverse mechanisms, such as apoptosis, pyroptosis, or necroptosis (Rius-Rocabert et al., 2020; Hu et al., 2022). OVs treatment enhance immune cell infiltration and promote inflammation within a highly immunosuppressed tumor microenvironment (TME), which might be critical in breaking the dysimmunity (Ribas et al., 2017; Hemminki et al., 2020). A diverse range of OVs is currently undergoing evaluation in both preclinical and clinical stages for the intervention of glioma, which includes GBM and LGG (Suryawanshi and Schulze, 2021; Li et al., 2022). Notably, a growing pool of highly potential OV candidates, such as adenovirus DNX-2401 (Gállego Pérez-Larraya et al., 2022) and adenovirus ICOVIR17 (Kiyokawa et al., 2021), are currently validating their potential to generate a sustained response in patients with malignant glioma in the postapproval trials (Hulou et al., 2016). The biological mechanisms responsible for OV treatment in glioma are not well understood, and there is a need to identify biomarkers that can offer new insights into treatment options.

In present study, we aimed at identifying the novel biomarkers in OV-infected glioma and providing improved opportunities for glioma diagnosis or prognosis. We carried out a thorough analysis utilizing publicly databases and online analysis tools to examine the impact of EV-A71 infection on gene ontology categories and pathways in glioma. Furthermore, we investigated the role of polypyrimidine tract binding protein 1 (PTBP1) as a prognostic factor in LGG and examined its association with clinicopathological characteristics and immune cell infiltration.

## Materials and methods

### Data sources and bioinformatical analysis

The mRNA expression data of EV-A71-infected glioma cells were obtained from the Boproject repository (PRJNA562271; https://www.ncbi.nlm.nih.gov/bioproject). Specifically, EV71 infection was infected in glioma cells (CCF-STTG1). To screen differentially expressed genes (DEGs), RNA-seq analysis was conducted using the Illumina HiSeqTM 2000 System. DEGs were identified between the EV-A71 infection group and the mock infection group based on their GFold values. The TCGA glioma datasets, which include LGG and GBM datasets, were analyzed using a bioinformatic tool GEPIA2.^1^ The genomic data of Chinese glioma patients was obtained from the CGGA database for this study (http://www.cgga.org.cn/index.jsp; Zhao et al., 2021). The protein expression images were obtained from the Human Protein Atlas (HPA, https://www.proteinatlas.org/). We acquired the distribution of the proteins across normal tissues and HCC tissues in the “Tissue” section and the “Pathology” section. The antibody utilized for performing IHC assay is CAB013507.

### Gene ontology and Kyoto Encyclopedia of Genes and Genomes analysis

To evaluate the functional classification, we utilized the DAVID Gene Functional Classification Tool (https://david.ncifcrf.gov/home.jsp; Sherman et al., 2022). The gene list of downregulated or upregulated DEGs was submitted, and we classified the extensive gene list into functional gene groups based on their relatedness.

### Survival analysis

To evaluate the association between PTBP1 level and poor prognosis, we conducted an analysis of both overall survival (OS) and disease-free survival (DFS). The glioma cases, encompassing both GBM and LGG patients, were stratified into the groups divided by the levels of PTBP1 TPM: a low PTBP1 TPM level group (bottom 50%) and a high PTBP1 TPM level group (top 50%). Kaplan–Meier curve was utilized to illustrate the survival status, and statistical significance was determined by setting a threshold of *p*-values less than 0.05.

### Immune cell infiltrating estimation

To investigate the association between TPBP1 level and immune cell infiltration in LGG and GBM cells, we utilized the ssGSEA and TIMER algorithms to analyze the TCGA data. The “gene module” available on the TIMER website was employed to visualize the correlation between TPBP1 level and immune infiltration in both GBM and LGG. Furthermore, we utilized the “Survival module” to examine the clinical relevance of immune infiltrates and assess their impact on survival differences. Additionally, we compared the levels of immune infiltrates in glioma with the presence of an IDH mutation using the “Mutation module.” Somatic copy number alterations (SCNAs) were defined into four groups by GISTIC 2.0. The SCNA module allows for the comparison of tumor infiltration levels between tumors with different somatic copy number alterations of PTBP1.

### Statistical analysis

Student’s t-tests or Wilcoxon rank-sum tests were used to detect significant differences in gene expression or immune cell enrichment. One-way ANOVA was used in analyzing gene expression in different cancer stage. Spearman rank correlation was utilized to analyze the correlation between PTBP1 and immune infiltration. A *p*-value of less than 0.05 was considered statistically significant. All data are reported as the mean ± standard error (SEM).

## Results

### Analysis of differential expression genes and gene oncology in entrovirus A71-infected glioma cells

**Figure 1:**
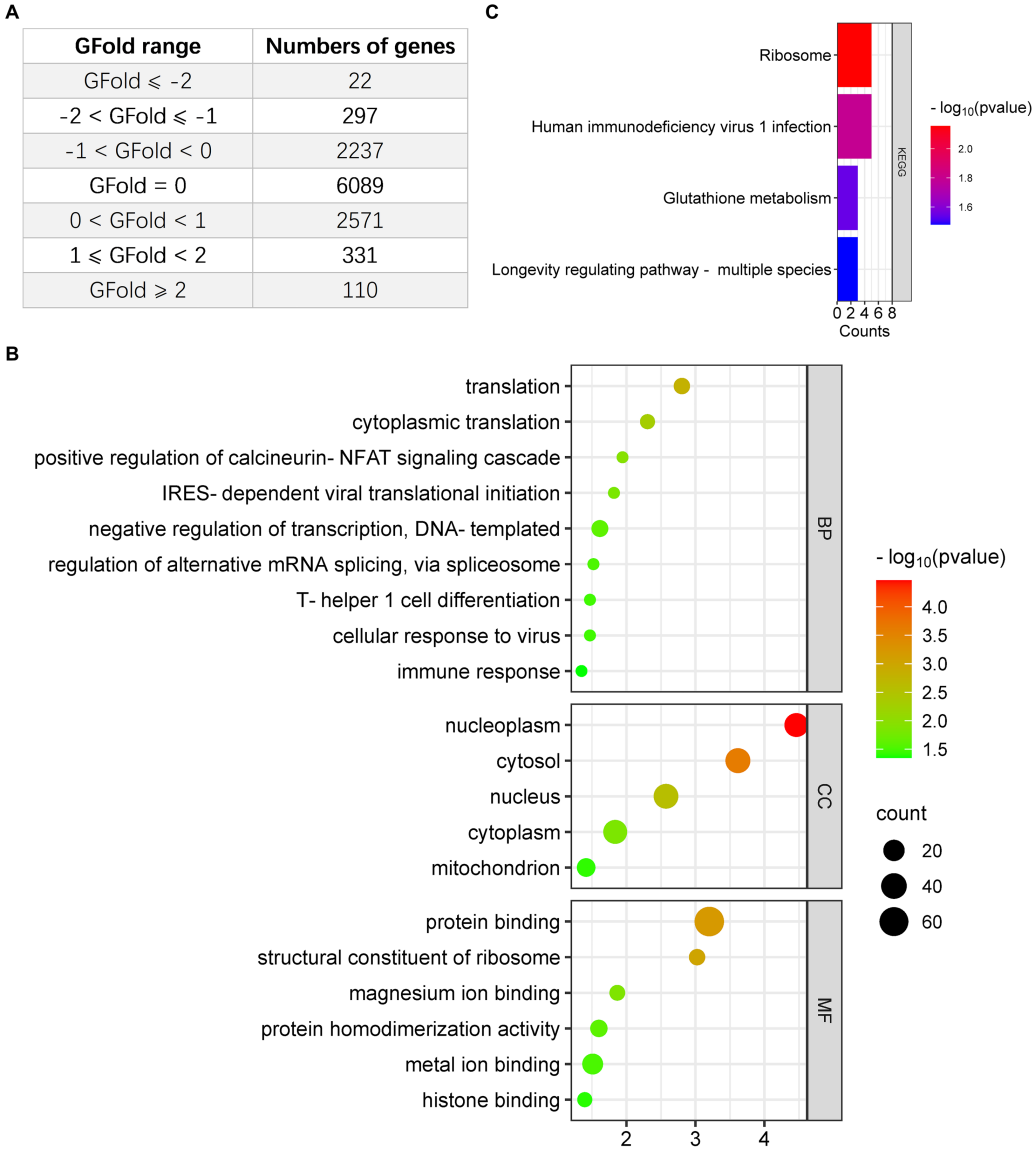
Analysis of downregulated DEGs in glioma cells infected with EV-A71, focusing on Gene Ontology (GO) and Kyoto Encyclopedia of Genes and Genomes (KEGG). **(A)** The count of DEGs identified in EV-A71-infected glioma cells. **(B)** BioPlanet provided annotations for the prominent Biological Process (BP), Molecular Function (MF), or Cellular Component (CC) categories associated with the downregulated DEGs following EV-A71 infection in glioma cells. **(C)** BioPlanet annotated the top four conserved pathways among the downregulated DEGs following EV-A71 infection.

Given the interconnection between oncolytic virotherapy and antitumor response in glioma (You et al., 2020; Zhang et al., 2020), we first examined the regulation of entrovirus (EV)-A71 infection on CCF-STTG1 cells. One dataset (PRJNA562271) obtained from Bioproject was included in this study: EV-A71 oncolysis of human malignant gliomas. Genes with GFold > −1 or GFold <1 in RNA-seq analysis were filtered out leaving total of 441 upregulated genes (Supplementary Table S1) and 319 downregulated genes to be analyzed in the research (Supplementary Table S2). Figure 1A shows the total number of DEGs in the comparisons EV-A71 infection vs. mock group. Our results show 22 down-regulated genes with GFold <= −2 and 297 genes with −2 <= GFold < −1, while 331 genes were up-regulated with 1 <= GFold < 2 and 110 genes were up-regulated with GFold >= 2 (Figure 1A). Based on the tumoricidal features of OV in glioma, we included 319 downregulated genes to identify the functional classification using DAVID online tool. According to the results of the functional GO enrichment analysis focusing on biological processes (BP), it was found that the 319 DEGs regulated a total of 9 functional categories. These categories were as follows: negative regulation of transcription (DNA-templated), translation, cytoplasmic translation, positive regulation of calcineurin-NFAT signaling cascade, IRES-dependent viral translational initiation, regulation of alternative mRNA splicing via spliceosome, cellular response to virus, T-helper 1 cell differentiation and immune response (Figure 1B). The KEGG enrichment analysis indicated that the downregulated DEGs showed associations with ribosome, human immunodeficiency virus 1 infection and glutathione metabolism (Figure 1C). Additionally, the result of GO and KEGG enrichment analysis with the 440 upregulated DEGs was illustrated in Supplementary Figure 1.

### Upregulation of PTBP1 in human glioma

**Figure 2:**
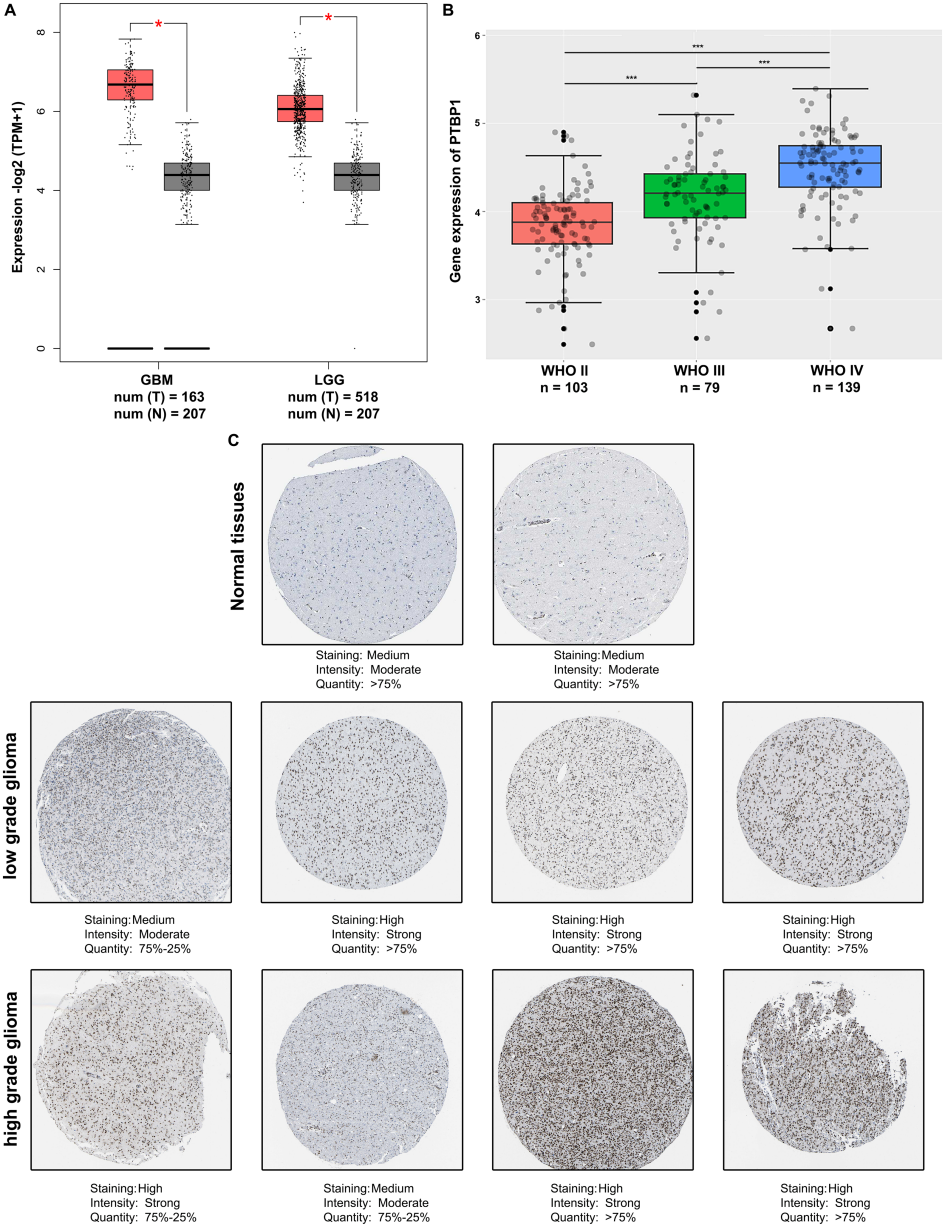
PTBP1 was upregulated in human glioma. **(A)** The expression levels of PTBP1 were validated by analyzing the TCGA data in LGG patients (*n* = 518), GBM patients (*n* = 207), and nontumor controls (*n* = 207). Statistical significance was indicated by *p < 0.05. **(B)** Analysis of CGGA data revealed the differential expression of PTBP1 mRNA across various glioma subgroups. Statistical significance was indicated by ***p < 0.001. **(C)** Representative images of glioma tissue specimens (LGG and GBM) with both low and high PTBP1 levels are presented, as quantified by IHC staining in HPA database.

Considering the involvement of PTBP1 in all categories of GO and KEGG enrichment analysis, we conducted an analysis using the publicly accessible TCGA database to examine the PTBP1 status in normal brain and glioma tissues. The findings from the TCGA database demonstrated a substantial increase in PTBP1 differential transcription levels among GBM (*n* = 163) or LGG (*n* = 518) samples when compared to normal (*n* = 207) samples (*p* < 0.05, Figure 2A). Additionally, the trends observed in TCGA data were also observed in CGGA dataset. Moreover, an RNA analysis conducted on Chinese human glioma tissues indicated a progressive increase in PTBP1 expression from WHO II to WHO IV stages, providing further confirmation of the distinctive PTBP1 status within glioma (*p* < 0.001, Figure 2B). Immunohistochemical staining was utilized to measure the protein levels of PTBP1 in human glioma samples. The analysis revealed a pronounced relative overexpression of PTBP1 in glioma tissues when compared to normal cerebral cortex tissues (Figure 2C). Moreover, a stronger intensity and more quantity of PTBP1 protein staining were found in high-grade glioma, compared with low-grade glioma (Figure 2C). These results indicated a positive correlation between PTBP1 level and the pathological stages of glioma, confirming the possibility involvement of PTBP1 in glioma advancement.

### Evaluation of PTBP1 as a prognostic biomarker in LGG

**Figure 3:**
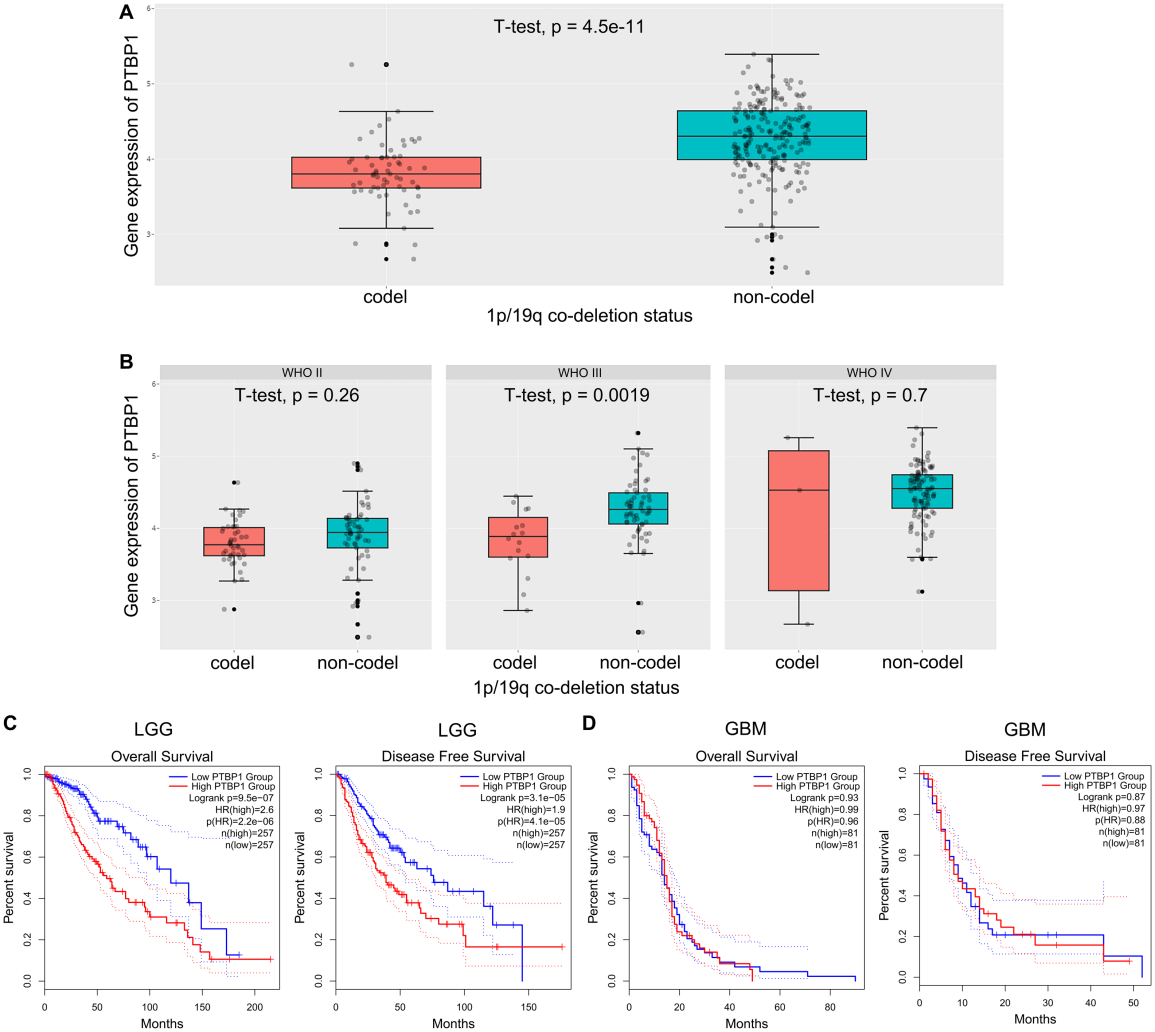
Correlation between PTBP1 mRNA expression and clinicopathological features in CGGA data. **(A)** The levels of PTBP1 mRNA expression were compared among glioma cases with different 1p/19q co-deletion statuses. **(B)** The levels of PTBP1 mRNA expression were further analyzed among different 1p/19q co-deletion statuses specifically in WHO II, WHO III, and WHO IV glioma. **(C,D)** The association between PTBP1 expression and survival was assessed among patients with LGG or GBM.

The co-deletion status of chromosomal 1p/19q represents a prominent driver in the development of glioma and is diffusely acknowledged as a robust prognostic gene in the study of gene mutations in glioma (Bhattacharya et al., 2021). Here, we explored whether 1p/19q co-deletion (codel) status may correlate with PTBP1 gene expression in glioma. In this regard, we acquired and examined the scaled gene expression profile from the CGGA dataset, arranging genes located on 1p/19q based on the genomic locations (Hu et al., 2017). We observed that in the total cohort of glioma cases, lower levels of PTBP1 significantly correlates with 1p/19q co-deletion (*p* = 4.5e-11, Figure 3A). Figure 3B shows that PTBP1 gene levels were obviously higher in non-codel group compared to codel group in the WHO III subtype (*p* = 0.0019). In both the WHO II and WHO IV subtypes, no statistically significant differences were observed in the expression of PTBP1 with respect to the 1p/19q co-deletion status (*p* > 0.05, Figure 3B). Multiple studies focusing on glioma mutations have consistently reported that the presence of 1p/19q co-deletion in glioma is related to a positive response, leading to improved survival rates (Barthel et al., 2019; Bhattacharya et al., 2021). Patients with high PTBP1 expression (cutoff-high: 50%) had a shorter OS of 60 months compared to 120 months in LGG patients with low PTBP1 level (cutoff-low: 50%; *p* < 0.001, HR 2.6, Figure 3C). Kaplan–Meier curves were shown in the right panel of Figure 3C, the median DFS was 40 months in patients with high PTBP1 expression, compared to 75 months in patients with low PTBP1 expression (*p* < 0.001, HR 1.9). However, neither OS or DFS were obviously related to PTBP1 levels in GBM (Figure 3D). Additionally, no statistically significant differences were observed between the groups based on PTBP1 expression status in terms of gender, age and progression status of glioma patients (Supplementary Figure 2; Table 1). Moreover, the areas under the curve (AUCs) of the time-dependent ROC curves were 0.973, indicating a high sensitivity and specificity of PTBP1 signature for predicting OS in glioma (Supplementary Figure 3).

**Table 1:** Characteristics of patients with glioma according to PTBP1 expression level.

Characteristics	Low expression of PTBP1	High expression of PTBP1	*p* value
*n*	349	350	
WHO grade, *n* (%)			< 0.001
G2	170 (26.7%)	54 (8.5%)	
G3	117 (18.4%)	128 (20.1%)	
G4	23 (3.6%)	145 (22.8%)	
IDH status, *n* (%)			< 0.001
WT	49 (7.1%)	197 (28.6%)	
Mut	295 (42.8%)	148 (21.5%)	
1p/19q codeletion, *n* (%)			< 0.001
Non-codel	222 (32.1%)	298 (43.1%)	
Codel	126 (18.2%)	46 (6.6%)	
Gender, *n* (%)			0.785
Female	147 (21%)	151 (21.6%)	
Male	202 (28.9%)	199 (28.5%)	
Age, *n* (%)			< 0.001
<= 60	310 (44.3%)	246 (35.2%)	
> 60	39 (5.6%)	104 (14.9%)	
Histological type, *n* (%)			< 0.001
Astrocytoma	109 (15.6%)	87 (12.4%)	
Oligoastrocytoma	86 (12.3%)	49 (7%)	
Oligodendroglioma	131 (18.7%)	69 (9.9%)	
Glioblastoma	23 (3.3%)	145 (20.7%)	
OS event, *n* (%)			< 0.001
Alive	278 (39.8%)	149 (21.3%)	
Dead	71 (10.2%)	201 (28.8%)	
DSS event, *n* (%)			< 0.001
No	279 (41.2%)	155 (22.9%)	
Yes	66 (9.7%)	178 (26.3%)	
Primary therapy outcome, *n* (%)			< 0.001
PD	49 (10.5%)	63 (13.5%)	
SD	93 (20%)	55 (11.8%)	
PR	42 (9%)	23 (4.9%)	
CR	96 (20.6%)	44 (9.5%)	

### Association of PTBP1 and immune infiltration characteristic in glioma

**Figure 4:**
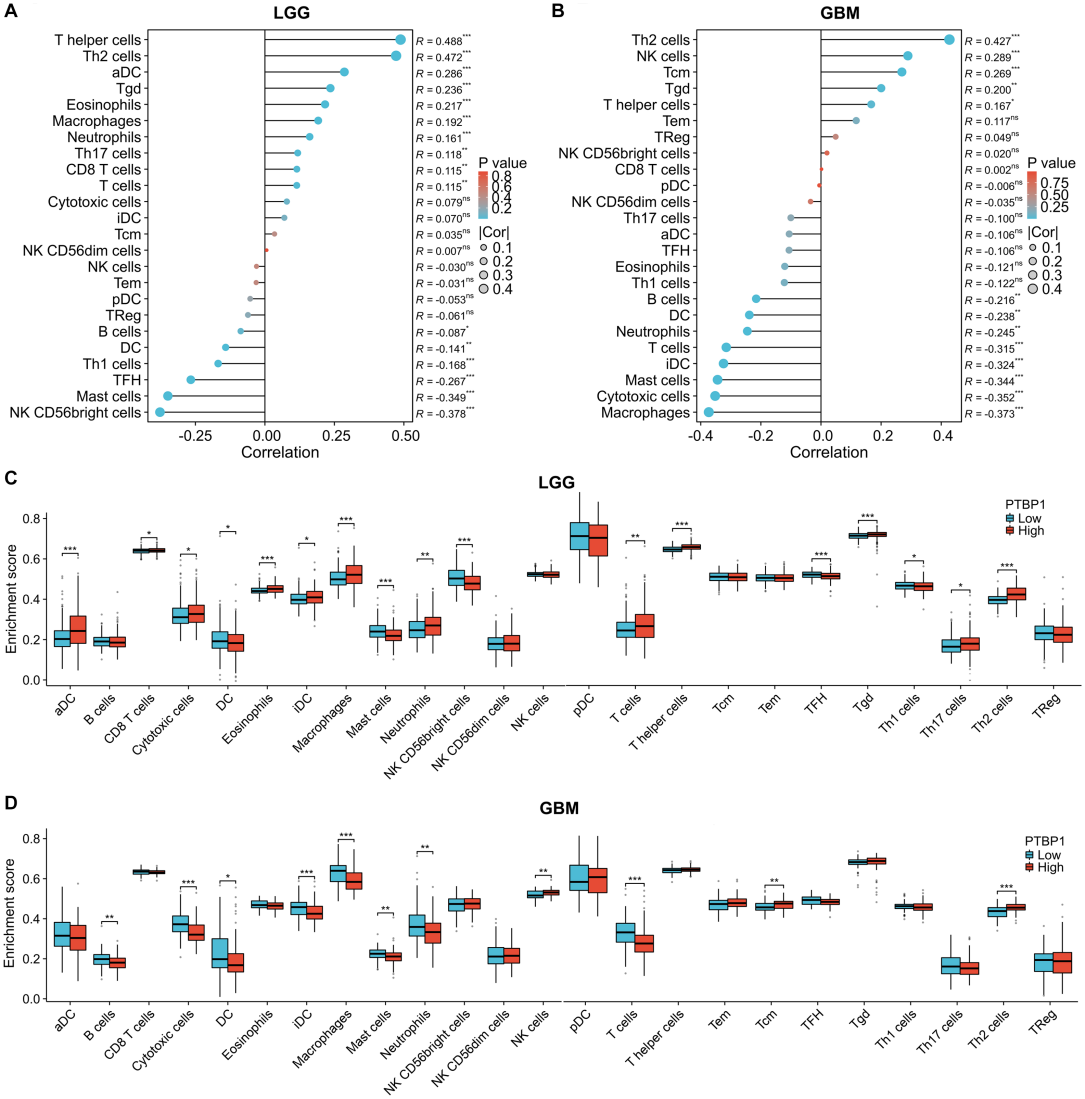
Association of PTBP1 expression and immune infiltration characteristic in glioma. To estimate immune cell infiltrations in TCGA data, the ssGESA algorithm was employed. Utilizing Pearson’s method, we determined the correlation between PTBP1 levels and immune cells in both **(A)** LGG and **(B)** GBM. Additionally, the enrichment score of immune cells was assessed in PTBP1low and PTBP1high samples, separately for **(C)** LGG and **(D)** GBM. Statistical significance was denoted by * for *p* < 0.05, ** for *p* < 0.01, and *** for *p* < 0.001.

OV therapy plays a significant role in stimulating the immune response against tumors, making it an essential component in eradicating cancer cells. To assess its potential anti-tumor efficacy, we subsequently evaluated the impact of PTBP1 expression on immune infiltration in gliomas using the ssGESA algorithm. As illustrated in Figures 4A,C, the correlation results implied that PTBP1 was linked with the presence of T helper cell (*p* < 0.001, R = 0.488), Th2 cells (*p* < 0.001, R = 0.472) and macrophages (*p* < 0.001, R = 0.192), but negatively linked with the presence of NK CD56bright cell (*p* < 0.001, R = −0.378), mast cell (*p* < 0.001, R = −0.349) and Tha1 cells (*p* < 0.001, R = −0.087). The correlation results, as depicted in Figures 4A,C, demonstrated significant associations between PTBP1 and the presence of T helper cells (*p* < 0.001, R = 0.488), Th2 cells (*p* < 0.001, R = 0.472), and macrophages (*p* < 0.001, R = 0.192) in LGG. However, there was a negative association between PTBP1 and the presence of NK CD56bright cells (*p* < 0.001, R = −0.378), mast cells (*p* < 0.001, R = −0.349), and Th1 cells (*p* < 0.001, R = −0.168) in LGG. The analysis of GBM samples presented in Figures 4B,D revealed significant associations between PTBP1 and the presence of Th2 cells (*p* < 0.001, R = 0.427) and NK cells (*p* < 0.001, R = 0.289). Conversely, we observed a negative correlation between PTBP1 expression and the presence of macrophages (*p* < 0.001, R = −0.373), cytotoxia cells (*p* < 0.001, R = −0.352), T cells (*p* < 0.001, R = −0.315), and T cells (*p* < 0.001, R = −0.315).

### Association of PTBP1 and unfavorable prognosis in LGG immune cells

**Figure 5:**
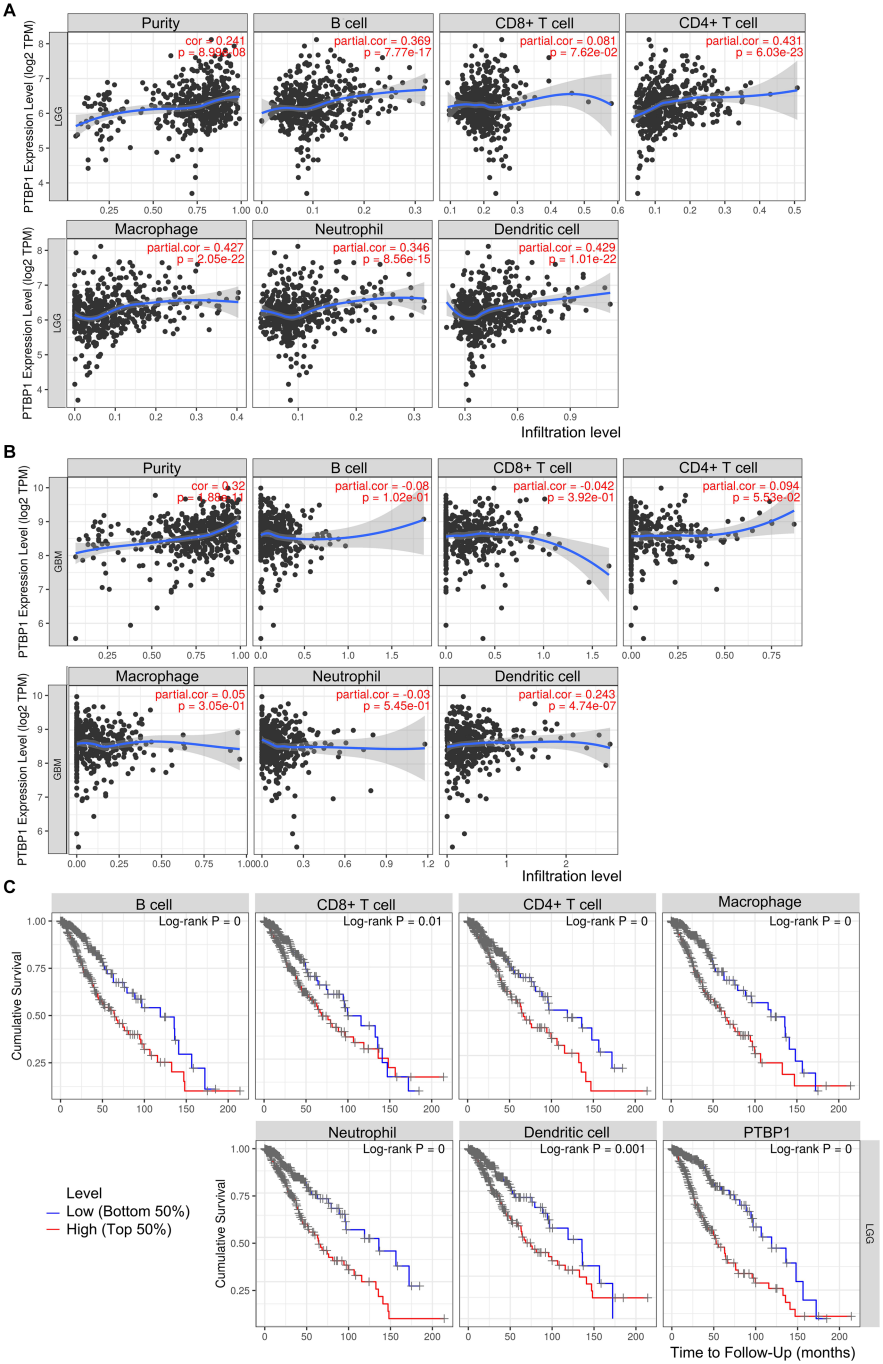
Association of PTBP1 expression and unfavorable prognosis in LGG immune cells. The correlation between PTBP1 levels and immune cells was analyzed in both **(A)** LGG and **(B)** GBM using TIMER algorithm. **(C)** Kaplan–Meier survival curves of OS comparing high (top 50%) and low (bottom 50%) immune infiltration in LGG.

**Figure 6:**
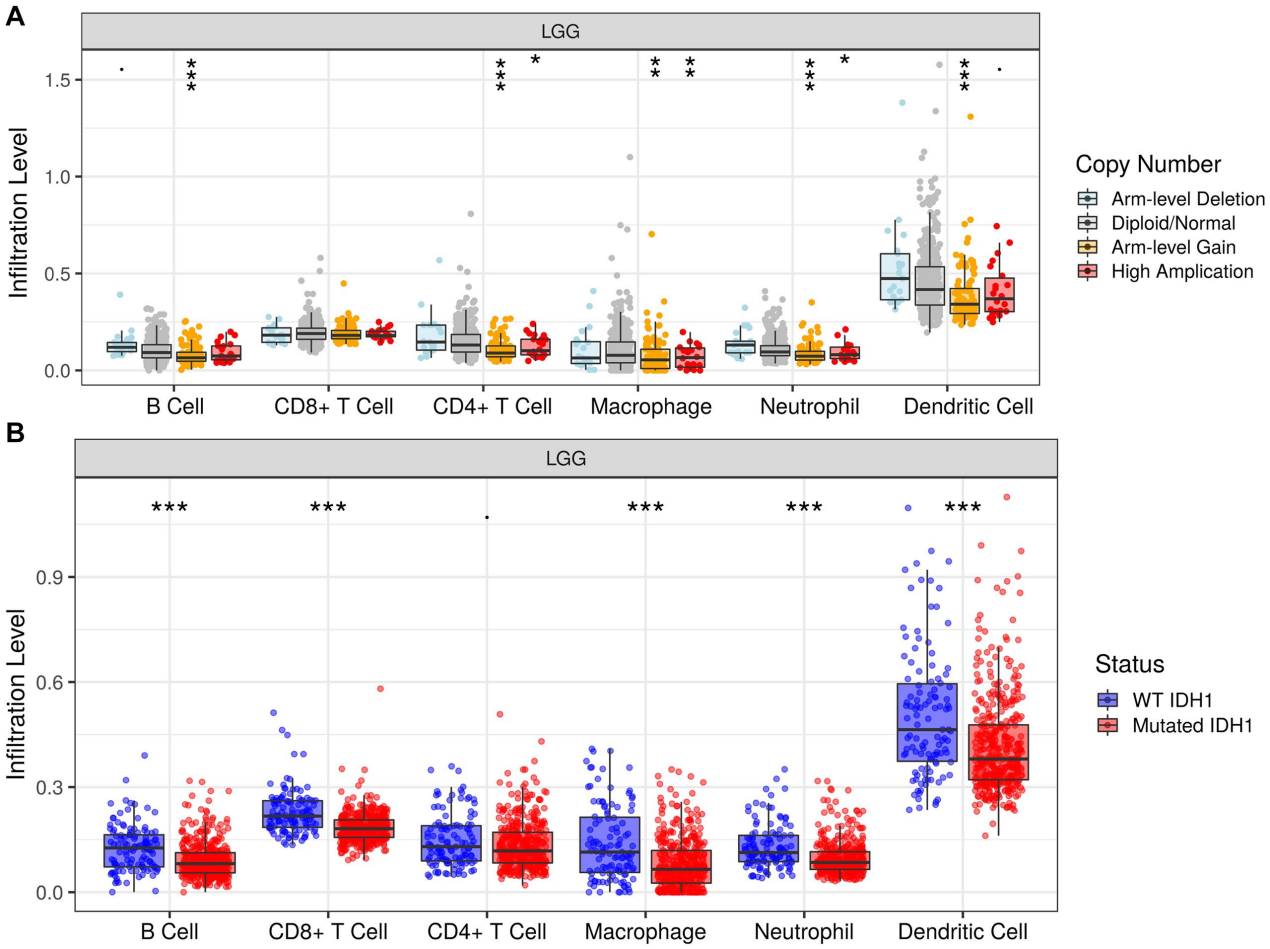
Association of infiltration levels and PTBP1 chromosomal change or IDH mutation in LGG cells. Histogram representing the infiltration levels of 6 immune cells (B cell, CD8 + T cell, CD4 + T cell, macrophage, neutrophil and dendritic cell) in different **(A)** copy number groups and **(B)** IDH mutation status.

In addition, we investigated the association between PTBP1 level and immune cell infiltration using the TIMER algorithm. The left-most panel of Figures 5A,B showed that highly expressed PTBP1 levels have positive association with tumor purity in LGG. The data depicted in Figure 5A indicated a positive correlation between PTBP1 levels and the infiltration of B cells (*p* = 7.77e-17, cor = 0.369), macrophages (*p* = 2.05e-22, cor = 0.427), and neutrophils (*p* = 8.56e-15, cor = 0.346) in LGG. This consistent finding was also observed using the ssGSEA computational tool. However, in GBM, no significant association was found between PTBP1 expression levels and immune cell infiltration (Figure 5B). Subsequently, we examined the correlation of immune infiltration with OS in LGG. Interestingly, similar to PTBP1 expression, an obvious correlation between high level of immune cell infiltration and worse overall survival (all *p*-values <0.001, Figure 5C). These findings suggest that PTBP1 may function as an independent adverse prognostic biomarker in LGG, potentially in relation to immune cell infiltration. Next, we investigated glioma-related chromosomal change or mutation to explore the potential mechanism in immune infiltration. SCNA module of TIMER showed the distributions of immune subset (B cells, CD4+ T cells, macrophage, neutrophil and dendritic cells) at arm-level gain or high amplification of PTBP1 (*p* < 0.05 or *p* < 0.001, Figure 6A). Moreover, mutation module of TIMER demonstrated that the levels of immune infiltrates were related to IDH mutation status in LGG (*p* < 0.001, Figure 6B).

## Discussion

The objective of this study was to examine the association between PTBP1 and EV-A71 infection in glioma cells, as well as evaluate the role of PTBP1 in patients diagnosed with LGG and GBM. The findings from our bioinformatic analysis demonstrated a substantial influence of PTBP1 expression on both survival and immune infiltration among LGG patients. These results indicate the potential clinical relevance of PTBP1 as a biomarker for patients with LGG.

The effect of oncolytic virotherapy in human glioma has been a subject of recent studies, generating a debate (Suryawanshi and Schulze, 2021). Although there is limited evidence supporting oncolytic virotherapy as a potential treatment for the challenges posed by the blood–brain barrier and the immunosuppressive TME, studies have suggested that ongoing clinical trials hold promise in establishing its satisfactory safety and significant immune response (Shoaf and Desjardins, 2022). Activation of the SCARB2/PMAIP1 axis has demonstrated considerable potential in facilitating cell oncolysis and, consequently, eradicating glioma cells (Zhang et al., 2020). In both *in vivo* and *in vitro* experiments, the presence of EV-A71 infection exerts a noteworthy influence in the anti-cancer process. Understanding the molecular networks governing glioma cells has been greatly enhanced by the mechanistic analysis of EV-A71 infection. In the present research, we conducted an evaluation of the DEGs associated with EV-A71 infection to identify potential regulatory processes or genes in glioma. By performing RNA-seq analysis, we observed a significant downregulation of DEGs in EV-A71-infected glioma cells. Importantly, our findings suggested a strong correlation between the downregulated DEGs and the cellular response to the virus, as well as the immune response in the infected glioma cells. These compelling findings prompted further investigations to unravel the mechanisms underlying EV-A71 infection in the regulation and molecular pathogenesis of glioma. Consequently, we shed light on the previously unexplored regulatory role of a top downregulated gene in glioma, which represents one of the hallmarks contributing to tumor progression.

PTBP1 is an RNA-binding protein with multifunctionality, involved in the regulatory process of mRNA splicing and apoptosis in numerous tumors and diseases (Liu et al., 2023). In a study by Gong et al., it was suggested that PTBP1, being upregulated in pan-cancer, plays a role in mediating oncogenesis and immunity by influencing the growth and cell cycle of osteosarcoma cells (Gong et al., 2022). According to previous studies, the downregulation of PTBP1 has been shown to impair immune surveillance, leading to the inhibition of inflammation-induced tumorigenesis (Georgilis et al., 2018). It is noteworthy to mention that inhibiting the expression of PTBP1 does not have an impact on the differentiation of glioblastoma cells, but contributes to the inhibition of cancer growth (Liu et al., 2022; Wang et al., 2022). A previous study has documented a connection between elevated PTBP1 expression levels and the WHO grade in glioma (Liu et al., 2022). Consistent with previous findings, our study revealed a significant correlation between PTBP1 level and advanced tumor stage at both the mRNA and protein expression in glioma. This observation indicates that PTBP1 may play a role in promoting the progression of glioma. In contrast to the unfavorable prognosis typically observed in GBM patients (Liu et al., 2022), these results revealed that high PTBP1 expression was not associated with worse prognosis in GBM. However, we observed that elevated PTBP1 expression was related to a worse prognosis in patients diagnosed with LGG.

Existing studies have documented the ability of OVs and the TME to generate immunostimulatory molecules, which play a crucial role in enabling the efficacy of immune therapies in the treatment of solid tumors (Sivanandam et al., 2019; Hemminki et al., 2020). The application of PD-1 blockade through OV infection has been reported to primarily result in enduring antitumor responses among cancer patients (Ribas et al., 2017). This approach is known to be correlated with the activation of the TME and increased infiltration of immune cells in melanoma (Ribas et al., 2017). An elevated level of immunostimulatory biomolecules is predominantly linked to the accumulation of T regulatory cells in GBM and is acknowledged for its role in compromising the immune system (Sokratous et al., 2017). In our study, we observed a relationship between TPBP1 and the infiltration of several immune cells in LGG. This association is likely to have contributed to the decline in OS. Furthermore, our investigation revealed a strong correlation between TPBP1 level and T helper cells, including CD4+ T cells and Th2 cells. The result suggests that TPBP1 might play a role in the unfavorable prognosis of LGG by interacting with T cells, specifically Th2 cells. The underlying reason for this finding is not yet fully understood; however, one potential explanation revolves around the equilibrium between Th1 and Th2 cells, which disrupts the dynamic balance of the cytokine network in humans and contributes to the initiation of tumor development (Romagnani, 1999; Matia-Garcia et al., 2021).

TME has gained prominence as a pivotal controller influencing the reaction to OV therapy. Owing to the distinct constitution of the extracellular matrix and immunologic surroundings within the cranial region, the brain’s TME exhibits distinct characteristics and multiple tumor prognostic markers. For example, EVA1C is thought to be associated with various immune markers, such as B cells and CD4+ T cells. This association indicates that two member of EVA family, EVA1B and EVA1C, might have a role in prognosticating elevated levels of immune infiltration within glioma (Hu and Qu, 2021; Qu et al., 2021b). In addition, a literature analyzed the immune-related signature in glioma and suggested that the expression of OLFML3 might additionally mirror an irregular immune condition (Qu et al., 2023). To gain insight into the mechanisms underlying the association between the risk score and immune cell infiltration, we performed a detailed analysis of the impact of somatic cell copy number alterations (CNAs) in TPBP1. Intriguingly, we found that arm-level deletion had a significant effect on the infiltration levels of B cells, CD4+ T cells, macrophages, and dendritic cells in LGG. The results provided compelling evidence of the pivotal regulatory role of TPBP1 in shaping the TME for patients with LGG.

It is important to note that our study has limitations, as it relied on publicly available data rather than clinical samples, and did not include evidence from *in vitro* and *in vivo* research. The significance of PTBP1 in EV-A71-infected glioma and its potential role in expression levels in glioma have been strongly suggested, highlighting the light spot of this study. As a continuation of this research, we will assess the molecular mechanism of PTBP1 following EV-A71 infection in glioma.

In general, this research contributes to the expanding body of evidence highlighting the significant involvement of EV-A71 infection in the gene regulation of glioma. Furthermore, our study demonstrated the potential prognostic significance of TPBP1 in LGG, as its overexpression was found to be linked to advanced tumor stage and a poorer prognosis. We also observed a correlation between TPBP1 and immune cell infiltration in LGG, a common occurrence within the immunosuppressive microenvironment of cancer. This study provides insight into a potential regulatory mechanism involved in OV treatment for glioma, laying the groundwork for future investigations aimed at unraveling the prospective molecular function of TPBP1 in glioma.

## Data availability statement

Publicly available datasets were analyzed in this study. This data can be found here: PRJNA562271, https://www.ncbi.nlm.nih.gov/bioproject.

## Ethics statement

Ethical approval was not required for the studies on humans in accordance with the local legislation and institutional requirements because only commercially available established cell lines were used.

## Author contributions

ZH, XY, and HD designed the conception of this study. ZH, XY, RD, and LC were involved in the data analysis. ZH and XY drafted the initial manuscript. The final version of the manuscript was reviewed and revised by RD, LC, CH, and HD. All authors contributed to the article and approved the submitted version.

## Footnotes

^1^ http://gepia2.cancer-pku.cn/#index

## Abbreviations

GBM: Glioblastoma multiforme
LGG: Low-grade glioma
OV: Oncolytic virus
TME: Tumor microenvironment
PTBP1: Polypyrimidine tract binding protein 1
DEG: Differentially expressed genes
GO: Gene ontology
KEGG: Kyoto Encyclopedia of Genes and Genomes
OS: Overall survival
DFS: Disease-free survival
SCNA: Somatic copy number alterations
EV: Entrovirus
BP: Biological processes
codel: Co-deletion

## References

[ref1] Barthel F. P. Johnson K. C. Varn F. S. Moskalik A. D. Tanner G. Kocakavuk E.. (2019). Longitudinal molecular trajectories of diffuse glioma in adults. Nature 576, 112–120. doi: 10.1038/s41586-019-1775-1, PMID: 31748746 PMC6897368

[ref2] Bhattacharya D. Sinha N. Saini J. (2021). Determining chromosomal arms 1p/19q co-deletion status in low graded glioma by cross correlation-periodogram pattern analysis. Sci. Rep. 11:23866. doi: 10.1038/s41598-021-03078-1, PMID: 34903768 PMC8668971

[ref3] Chen R. Smith-Cohn M. Cohen A. L. Colman H. (2017). Glioma subclassifications and their clinical significance. Neurotherapeutics 14, 284–297. doi: 10.1007/s13311-017-0519-x, PMID: 28281173 PMC5398991

[ref4] Gállego Pérez-Larraya J. Garcia-Moure M. Labiano S. Patiño-García A. Dobbs J. Gonzalez-Huarriz M.. (2022). Oncolytic DNX-2401 virus for pediatric diffuse intrinsic pontine glioma. N. Engl. J. Med. 386, 2471–2481. doi: 10.1056/NEJMoa2202028, PMID: 35767439

[ref5] Georgilis A. Klotz S. Hanley C. J. Herranz N. Weirich B. Morancho B.. (2018). PTBP1-mediated alternative splicing regulates the inflammatory Secretome and the pro-tumorigenic effects of senescent cells. Cancer Cell 34, 85–102.e9. doi: 10.1016/j.ccell.2018.06.007, PMID: 29990503 PMC6048363

[ref6] Gong H. Jiang A. Jiang R. Wang Y. Zhang D. Wu Z.. (2022). PTBP1 as a promising predictor of poor prognosis by regulating cell proliferation, immunosuppression, and drug sensitivity in SARC. Oxidative Med. Cell. Longev. 2022, 1–26. doi: 10.1155/2022/5687238 PMC9151003 35651729

[ref7] Hemminki O. Dos Santos J. M. Hemminki A. (2020). Oncolytic viruses for cancer immunotherapy. J. Hematol. Oncol. 13:84. doi: 10.1186/s13045-020-00922-1, PMID: 32600470 PMC7325106

[ref8] Hu X. Martinez-Ledesma E. Zheng S. Kim H. Barthel F. Jiang T.. (2017). Multigene signature for predicting prognosis of patients with 1p19q co-deletion diffuse glioma. Neuro-Oncology 19, 786–795. doi: 10.1093/neuonc/now285, PMID: 28340142 PMC5464432

[ref9] Hu Z. Qu S. (2021). EVA1C is a potential prognostic biomarker and correlated with immune infiltration levels in WHO grade II/III glioma. Front. Immunol. 12:683572. doi: 10.3389/fimmu.2021.683572, PMID: 34267752 PMC8277382

[ref10] Hu M. Wang B. Wu C. (2022). Editorial: the role of DNA viruses in human cancers. Front. Cell. Infect. Microbiol. 12:1103505. doi: 10.3389/fcimb.2022.1103505, PMID: 36569205 PMC9773820

[ref11] Hu M. Xing B. Yang M. Han R. Pan H. Guo H.. (2023). Characterization of a novel genus of jumbo phages and their application in wastewater treatment. iScience 26:106947. doi: 10.1016/j.isci.2023.106947, PMID: 37324530 PMC10265529

[ref12] Hulou M. M. Cho C. F. Chiocca E. A. Bjerkvig R. (2016). Experimental therapies: gene therapies and oncolytic viruses. Handb. Clin. Neurol. 134, 183–197. doi: 10.1016/B978-0-12-802997-8.00011-6 26948355

[ref13] Kiyokawa J. Kawamura Y. Ghouse S. M. Acar S. Barçın E. Martínez-Quintanilla J.. (2021). Modification of extracellular matrix enhances oncolytic adenovirus immunotherapy in glioblastoma. Clin. Cancer Res. 27, 889–902. doi: 10.1158/1078-0432.CCR-20-2400, PMID: 33257429 PMC7854507

[ref14] Le Rhun E. Preusser M. Roth P. Reardon D. A. van den Bent M. Wen P.. (2019). Molecular targeted therapy of glioblastoma. Cancer Treat. Rev. 80:101896. doi: 10.1016/j.ctrv.2019.101896 31541850

[ref15] Li J. Meng Q. Zhou X. Zhao H. Wang K. Niu H.. (2022). Gospel of malignant glioma: oncolytic virus therapy. Gene 818:146217. doi: 10.1016/j.gene.2022.146217, PMID: 35093451

[ref16] Liu P. He G. C. Tan Y. Z. Liu G. X. Liu A. M. Zhu X. P.. (2022). PTBP1 is a novel poor prognostic factor for glioma. Bio. Med. Res. Int. 2022, 1–11. doi: 10.1155/2022/7590997 PMC8923792 35299889

[ref17] Liu H. L. Lu X. M. Wang H. Y. Hu K. B. Wu Q. Y. Liao P.. (2023). The role of RNA splicing factor PTBP1 in neuronal development. Biochim. Biophys. Acta, Mol. Cell Res. 1870:119506. doi: 10.1016/j.bbamcr.2023.119506, PMID: 37263298

[ref18] Malik N. Geraghty B. Dasgupta A. Maralani P. J. Sandhu M. Detsky J.. (2021). MRI radiomics to differentiate between low grade glioma and glioblastoma peritumoral region. J. Neuro-Oncol. 155, 181–191. doi: 10.1007/s11060-021-03866-9, PMID: 34694564

[ref19] Matia-Garcia I. Vadillo E. Pelayo R. Muñoz-Valle J. F. García-Chagollán M. Loaeza-Loaeza J.. (2021). Th1/Th2 balance in young subjects: relationship with cytokine levels and metabolic profile. J. Inflamm. Res. 14, 6587–6600. doi: 10.2147/JIR.S342545, PMID: 34908860 PMC8664383

[ref20] Qu S. Chen Z. Liu B. Liu J. Wang H. (2021a). N6-methyladenine-related genes affect biological behavior and the prognosis of glioma. Cancer Med. 10, 98–108. doi: 10.1002/cam4.3574, PMID: 33264518 PMC7826482

[ref21] Qu S. Huang C. Zhu T. Wang K. Zhang H. Wang L.. (2023). OLFML3, as a potential predictor of prognosis and therapeutic target for glioma, is closely related to immune cell infiltration. VIEW 4:20220052. doi: 10.1002/VIW.20220052

[ref22] Qu S. Liu S. Qiu W. Liu J. Wang H. (2020). Screening of autophagy genes as prognostic indicators for glioma patients. Am. J. Transl. Res. 12, 5320–5331. PMID: 33042422 PMC7540153

[ref23] Qu S. Liu J. Wang H. (2021b). EVA1B to evaluate the tumor immune microenvironment and clinical prognosis in glioma. Front. Immunol. 12:648416. doi: 10.3389/fimmu.2021.648416, PMID: 33889156 PMC8056259

[ref24] Ribas A. Dummer R. Puzanov I. Vander Walde A. Andtbacka R. H. I. Michielin O.. (2017). Oncolytic virotherapy promotes intratumoral T cell infiltration and improves anti-PD-1 immunotherapy. Cells 170, 1109–19.e10. doi: 10.1016/j.cell.2017.08.027, PMID: 28886381 PMC8034392

[ref25] Rius-Rocabert S. García-Romero N. García A. Ayuso-Sacido A. Nistal-Villan E. (2020). Oncolytic Virotherapy in glioma tumors. Int. J. Mol. Sci. 21:7604. doi: 10.3390/ijms21207604, PMID: 33066689 PMC7589679

[ref26] Romagnani S. (1999). Th1/Th2 cells. Inflamm. Bowel Dis. 5, 285–294. doi: 10.1097/00054725-199911000-00009 10579123

[ref27] Russell S. J. Peng K. W. Bell J. C. (2012). Oncolytic virotherapy. Nat. Biotechnol. 30, 658–670. doi: 10.1038/nbt.2287, PMID: 22781695 PMC3888062

[ref28] Sherman B. T. Hao M. Qiu J. Jiao X. Baseler M. W. Lane H. C.. (2022). DAVID: a web server for functional enrichment analysis and functional annotation of gene lists (2021 update). Nucleic Acids Res. 50, W216–W221. doi: 10.1093/nar/gkac194, PMID: 35325185 PMC9252805

[ref29] Shoaf M. L. Desjardins A. (2022). Oncolytic viral therapy for malignant glioma and their application in clinical practice. Neurotherapeutics 19, 1818–1831. doi: 10.1007/s13311-022-01256-1, PMID: 35674873 PMC9723031

[ref30] Sivanandam V. LaRocca C. J. Chen N. G. Fong Y. Warner S. G. (2019). Oncolytic viruses and immune checkpoint inhibition: the best of both worlds. Mol. Therapy Oncolytics 13, 93–106. doi: 10.1016/j.omto.2019.04.003, PMID: 31080879 PMC6503136

[ref31] Sokratous G. Polyzoidis S. Ashkan K. (2017). Immune infiltration of tumor microenvironment following immunotherapy for glioblastoma multiforme. Hum. Vaccin. Immunother. 13, 2575–2582. doi: 10.1080/21645515.2017.1303582, PMID: 28362548 PMC5703406

[ref32] Suryawanshi Y. R. Schulze A. J. (2021). Oncolytic viruses for malignant glioma: on the verge of success? Viruses 13:1294. doi: 10.3390/v13071294, PMID: 34372501 PMC8310195

[ref33] van Tellingen O. Yetkin-Arik B. de Gooijer M. C. Wesseling P. Wurdinger T. de Vries H. E. (2015). Overcoming the blood-brain tumor barrier for effective glioblastoma treatment. Drug Resist. Updat. 19, 1–12. doi: 10.1016/j.drup.2015.02.002 25791797

[ref34] Wang K. Pan S. Zhao P. Liu L. Chen Z. Bao H.. (2022). PTBP1 knockdown promotes neural differentiation of glioblastoma cells through UNC5B receptor. Theranostics 12, 3847–3861. doi: 10.7150/thno.71100, PMID: 35664063 PMC9131277

[ref35] Xia J. Li S. Liu S. Zhang L. (2023). Aldehyde dehydrogenase in solid tumors and other diseases: potential biomarkers and therapeutic targets. Media Commun. 4:e195. doi: 10.1002/mco2.195 PMC9842923 36694633

[ref36] You L. Chen J. Liu W. Xiang Q. Luo Z. Wang W.. (2020). Enterovirus 71 induces neural cell apoptosis and autophagy through promoting ACOX1 downregulation and ROS generation. Virulence 11, 537–553. doi: 10.1080/21505594.2020.1766790, PMID: 32434419 PMC7250321

[ref37] Zhang X. Wang H. Sun Y. Qi M. Li W. Zhang Z.. (2020). Enterovirus A71 Oncolysis of malignant gliomas. Mol. Ther. 28, 1533–1546. doi: 10.1016/j.ymthe.2020.04.005, PMID: 32304669 PMC7264442

[ref38] Zhao Z. Zhang K.-N. Wang Q. Li G. Zeng F. Zhang Y.. (2021). Chinese glioma genome atlas (CGGA): a comprehensive resource with functional genomic data from Chinese glioma patients. Genom. Prot. Bioinform. 19, 1–12. doi: 10.1016/j.gpb.2020.10.005, PMID: 33662628 PMC8498921

