# Supplementary material for: A novel oncolytic virus-based biomarker participates in prognosis and tumor immune infiltration of glioma
Source: Front Microbiol. 2023 Sep 22;14:1249289. doi: 10.3389/fmicb.2023.1249289 (PMC10556503; doi:10.3389/fmicb.2023.1249289)
Supplement: Supplementary file 1 [file Table_1.pdf]

Supplementary Table S1 A total of upregulated DEGs in EV-A71-infected glioma

GeneSymbol	GFOLD(0.01)	log2fdc	regulation
RNF152	1.50504	2.07339	up
CLIC4	1.50587	1.65795	up
SLC38A2	1.51347	1.7991	up
SPAG1	1.51353	2.7146	up
ADM	1.51753	1.8205	up
OGFR	1.52142	1.8573	up
PLK2	1.53092	1.70059	up
CDT1	1.53734	1.86547	up
RAB30	1.53888	3.35028	up
ZNF442	1.53888	3.35028	up
SLC25A28	1.54332	1.89748	up
BCAR3	1.55009	1.9232	up
ZNF134	1.55733	2.12933	up
FAM176A	1.56191	1.8731	up
PNPT1	1.57138	1.85026	up
LAP3	1.58537	1.71712	up
XAF1	1.59088	2.40687	up
ZBTB43	1.59697	2.07408	up
NFAT5	1.59741	2.00236	up
VEGFC	1.60558	1.81134	up
MIR100HG	1.60861	2.13768	up
LAMC2	1.60906	2.92366	up
KYNU	1.61005	1.6929	up
NNMT	1.61143	1.73139	up
MCL1	1.61272	1.84146	up
FLOT1	1.61727	1.71867	up
MX2	1.61898	2.04772	up
SLC25A37	1.62702	2.09208	up
PIM1	1.62938	1.89794	up
STK10	1.63731	2.30422	up
RPS8	1.63904	1.69573	up
MAP2K3	1.64066	2.26867	up
CLK1	1.64411	1.8497	up
RHEBL1	1.64681	3.33579	up
MCM7	1.64904	1.76064	up
ADORA2A	1.65041	3.00411	up
ZNF121	1.65766	3.25487	up
HERPUD1	1.66212	1.93585	up
GBP3	1.66653	1.87083	up
TIPARP	1.66899	1.96075	up
DCP1A	1.67	2.19623	up
TGIF1	1.6751	1.99344	up

JAK2	1.67592	2.14605	up
F3	1.6773	1.9556	up
UBXN7	1.68819	2.28134	up
IL11	1.69199	2.87635	up
YPEL2	1.69199	2.87635	up
ARRDC2	1.69321	2.24017	up
KLF4	1.697	2.25577	up
ZNF267	1.7176	2.14263	up
NFKB1	1.73398	1.98977	up
CCNL1	1.73489	2.06426	up
IRF2	1.74441	2.01546	up
C17orf44	1.74697	2.46827	up
GADD45A	1.75008	2.03332	up
STK17B	1.76696	2.10042	up
ID2	1.76754	1.91272	up
MAP3K8	1.76926	2.59625	up
FTH1	1.77389	1.80685	up
ZNFX1	1.77718	2.04882	up
EHD1	1.79407	2.03746	up
SLC2A3	1.7942	2.29139	up
GDF15	1.79672	2.1415	up
PRELP	1.80038	2.58262	up
JUN	1.80417	2.00796	up
PARP14	1.80447	2.14346	up
SPRY2	1.80468	2.52272	up
MDM2	1.80658	1.83134	up
OAS2	1.81255	2.14731	up
FMO5	1.81959	2.75082	up
CDKN2B	1.83152	2.31058	up
SMAD7	1.83553	2.27033	up
WTAP	1.8505	2.01587	up
RHOC	1.86074	2.01487	up
HERC6	1.87093	2.47071	up
TP63	1.89802	3.22999	up
DUSP1	1.89925	2.62638	up
TRIM26	1.90855	2.11973	up
NFKBIE	1.90978	2.40687	up
C21orf91	1.90988	2.35748	up
DUSP8	1.91544	3.57679	up
PSMA2	1.91992	2.05252	up
SPHK1	1.92855	2.37231	up
SPSB1	1.94448	2.24847	up
FAM65B	1.95477	2.87635	up
OTUD4	1.95892	2.34961	up

IFI44L	1.96881	2.26929	up
JHDM1D	1.99092	3.27324	up
TNFAIP6	2.006	3.06398	up
NEAT1	2.00818	2.10112	up
EPSTI1	2.01932	2.53122	up
PML	2.03608	2.29317	up
C8orf4	2.03672	2.22948	up
ZNF20	2.03703	2.84778	up
RIPK2	2.05072	2.34107	up
ADPRHL2	2.05242	2.34361	up
HIVEP2	2.0602	2.71641	up
PTPN12	2.06118	2.32739	up
C1QTNF1	2.07544	2.29394	up
GBP2	2.07948	2.34332	up
STAT5A	2.08704	2.5691	up
FAM46A	2.08917	2.36334	up
PDCD1LG2	2.11126	2.87635	up
EIF2C2	2.13311	3.18216	up
ZNF44	2.1507	2.91383	up
GADD45B	2.15906	2.49302	up
IRF1	2.16287	2.47451	up
TRIP10	2.16847	2.5715	up
IFIT5	2.17105	2.43058	up
SP110	2.17226	2.61797	up
ZCCHC2	2.18021	2.64338	up
EGR1	2.18873	2.55899	up
CMPK2	2.2025	2.60218	up
GFPT2	2.20374	2.44246	up
IFNGR2	2.2161	2.67084	up
GBP5	2.21754	3.26102	up
SAMD9	2.22044	2.55031	up
MFSD2A	2.23523	3.70643	up
EDN1	2.26062	3.52405	up
IRF7	2.26608	2.5556	up
OAS1	2.26747	2.69177	up
NAV3	2.28595	2.76588	up
ERRFI1	2.29486	2.62856	up
STK40	2.3086	2.68371	up
ARL5B	2.31247	3.21196	up
DKK1	2.3127	3.85211	up
CCRN4L	2.33243	2.95697	up
MT2A	2.3393	2.42211	up
IL15RA	2.34336	2.71766	up
RELB	2.35028	2.84159	up

TNFSF13B	2.3619	3.66062	up
SAMHD1	2.36764	2.96382	up
ZNF496	2.3697	4.09875	up
IL12A	2.43634	2.90777	up
IL24	2.43888	3.96946	up
SLC16A6	2.44231	4.16586	up
ISG20	2.46011	3.91588	up
FST	2.53486	3.18341	up
ZNF844	2.53784	4.14937	up
ITPRIP	2.54049	3.05113	up
NAMPT	2.54539	2.80735	up
PPP1R15A	2.56902	2.91359	up
TBX2	2.5914	3.61332	up
USP18	2.60167	2.87072	up
MX1	2.61877	3.06641	up
HBEGF	2.62382	4.0076	up
BDKRB2	2.62678	2.94483	up
DDX58	2.63703	3.08786	up
SAMD4A	2.68482	3.03895	up
IFI44	2.70504	2.89401	up
RCAN1	2.72576	2.93171	up
XBP1	2.78117	2.90724	up
CH25H	2.81312	4.08725	up
CCL5	2.81497	4.51378	up
CTGF	2.83496	3.1797	up
ZSWIM4	2.87246	4.14383	up
NINJ1	2.91395	3.12006	up
ARRDC3	2.9586	3.23649	up
NCOA7	2.96432	3.27236	up
CCL8	2.99418	4.49302	up
BBC3	3.02557	3.63885	up
ICAM1	3.03121	4.25487	up
SLC39A14	3.14406	3.42599	up
C11orf96	3.16278	3.88895	up
VCAM1	3.18775	3.29171	up
IFIH1	3.19997	3.63275	up
GCH1	3.20121	3.96548	up
ADAP1	3.23551	4.15319	up
ZC3H12A	3.27735	3.71589	up
IRAK2	3.31163	4.66395	up
NFKBIA	3.33643	3.50711	up
-	3.38992	4.20468	up
NGFR	3.52317	5.08581	up
BIRC3	3.53348	4.01871	up

-	3.63644	4.4452	up
TNFAIP2	3.66225	3.90067	up
SOD2	3.70369	3.82299	up
CCL2	3.86429	3.93527	up
CYR61	3.99167	4.22102	up
HERC5	4.08573	5.54689	up
LIF	4.22266	4.50932	up
ZC3HAV1	4.23174	4.42906	up
PMAIP1	4.42366	4.81869	up
NFKBIZ	4.45357	4.98707	up
IFIT1	4.61664	4.85969	up
NFKB2	4.62518	5.11995	up
TNFAIP3	4.89655	6.27248	up
RND3	5.09407	5.79166	up
OASL	5.12103	5.63653	up
-	5.27762	6.06398	up
ATF3	5.3327	6.70361	up
RSAD2	5.55895	6.00159	up
PTGS2	5.7612	6.01188	up
IFIT3	5.85119	6.24559	up
IFIT2	6.9771	7.31911	up
IL6	7.3346	8.00804	up
CXCL3	8.67916	10.289	up
IL8	8.8565	9.5139	up
