# Supplementary material for: A novel oncolytic virus-based biomarker participates in prognosis and tumor immune infiltration of glioma
Source: Front Microbiol. 2023 Sep 22;14:1249289. doi: 10.3389/fmicb.2023.1249289 (PMC10556503; doi:10.3389/fmicb.2023.1249289)
Supplement: Supplementary file 2 [file Table_2.pdf]

Supplementary Table S2 A total of downregulated DEGs in EV-A71-infected glioma

GeneSymbol	GFOLD (0.01)	log2fdc	regulation
HSPA2	-2.8202	-3.09248	down
PPP1R3C	-2.71197	-3.38873	down
HS6ST1	-2.70418	-2.94216	down
PGRMC1	-2.60448	-2.99154	down
ABCD3	-2.46471	-2.94844	down
NDUFS7	-2.45165	-2.83629	down
RALGDS	-2.4268	-3.25293	down
AP1S1	-2.36281	-2.74203	down
WDR67	-2.31209	-4.00629	down
THAP7	-2.23459	-2.70507	down
GDF5	-2.19526	-2.79607	down
TIGD5	-2.19121	-2.99401	down
CALM2	-2.10741	-2.23901	down
ARHGAP33	-2.10522	-2.70861	down
SIGMAR1	-2.10375	-2.42727	down
CDC42EP1	-2.07845	-2.33539	down
MRPL16	-2.06935	-2.53528	down
UFC1	-2.05988	-2.26026	down
ALKBH7	-2.05711	-2.45626	down
ZBTB4	-2.03706	-2.47768	down
RPS20	-2.0309	-2.11737	down
MVP	-2.01132	-2.22554	down
APOBEC3B	-1.99283	-2.30101	down
CASD1	-1.97583	-2.52138	down
PLA2G16	-1.94958	-2.54883	down
CNO	-1.94251	-2.67208	down
ZYG11B	-1.92849	-2.28596	down
PTBP1	-1.91135	-2.09173	down
DGKD	-1.88926	-2.50884	down
C13orf15	-1.87778	-2.07517	down

NLRX1	-1.85235	-2.51596	down
MLLT11	-1.8452	-2.4479	down
MRPL49	-1.82532	-2.35668	down
PI15	-1.80828	-2.06037	down
SCAND2	-1.80482	-2.83789	down
ZWINT	-1.77638	-2.01986	down
RPL22	-1.77134	-1.91838	down
PSMG3	-1.77025	-2.08365	down
PAK4	-1.76042	-2.40493	down
ZNF322A	-1.75344	-3.10093	down
PARG	-1.74595	-2.24259	down
LIMCH1	-1.74338	-2.03637	down
TOP2A	-1.74022	-1.88562	down
ZNF323	-1.74005	-2.32955	down
XPO6	-1.72515	-2.03269	down
GPX1	-1.72159	-1.87176	down
CSTF1	-1.71828	-2.30063	down
BIVM	-1.71341	-2.5699	down
PIF1	-1.70252	-2.44196	down
SNRPD3	-1.68714	-2.02279	down
POLR3GL	-1.67903	-2.13603	down
LOXL1	-1.67846	-1.80986	down
IER5L	-1.67777	-2.50302	down
IL10RB	-1.6721	-2.08558	down
HEXIM1	-1.66834	-2.37846	down
EZH1	-1.6553	-2.4335	down
C17orf79	-1.65444	-2.02733	down
P2RX6	-1.65254	-2.26221	down
SAMM50	-1.64297	-2.09743	down
INTS9	-1.62776	-2.89448	down
C7orf47	-1.6164	-1.94026	down

CDCA7	-1.60887	-2.10513	down
UQCRQ	-1.60682	-1.7294	down
RANBP9	-1.60415	-1.9619	down
RPL38	-1.60256	-1.70036	down
CPSF3	-1.59905	-1.92584	down
FAM160B2	-1.5975	-2.03967	down
FOXG1	-1.59286	-1.89674	down
TSHZ1	-1.58771	-3.3331	down
AZI1	-1.58464	-2.17991	down
IDH1	-1.58347	-1.99154	down
PDXP	-1.57756	-2.14301	down
PGLS	-1.56671	-1.84194	down
TWSG1	-1.5627	-1.87144	down
C7orf11	-1.56091	-1.97748	down
MTA3	-1.55489	-2.31866	down
PDE5A	-1.55376	-1.649	down
LOC253039	-1.55173	-3.03054	down
GCLC	-1.54787	-1.86596	down
MOBKL2A	-1.54303	-2.13995	down
RNF40	-1.54085	-1.98803	down
HIRA	-1.5381	-2.2145	down
HRAS	-1.5364	-1.80148	down
HMGN1	-1.52661	-1.68275	down
AKT1S1	-1.52327	-1.98505	down
RNF20	-1.51143	-2.118	down
ZNF32	-1.51077	-1.99685	down
RPL41	-1.50425	-1.57279	down
