# Supplementary figures and images for: A novel oncolytic virus-based biomarker participates in prognosis and tumor immune infiltration of glioma
Source: Front Microbiol. 2023 Sep 22;14:1249289. doi: 10.3389/fmicb.2023.1249289 (PMC10556503; doi:10.3389/fmicb.2023.1249289)

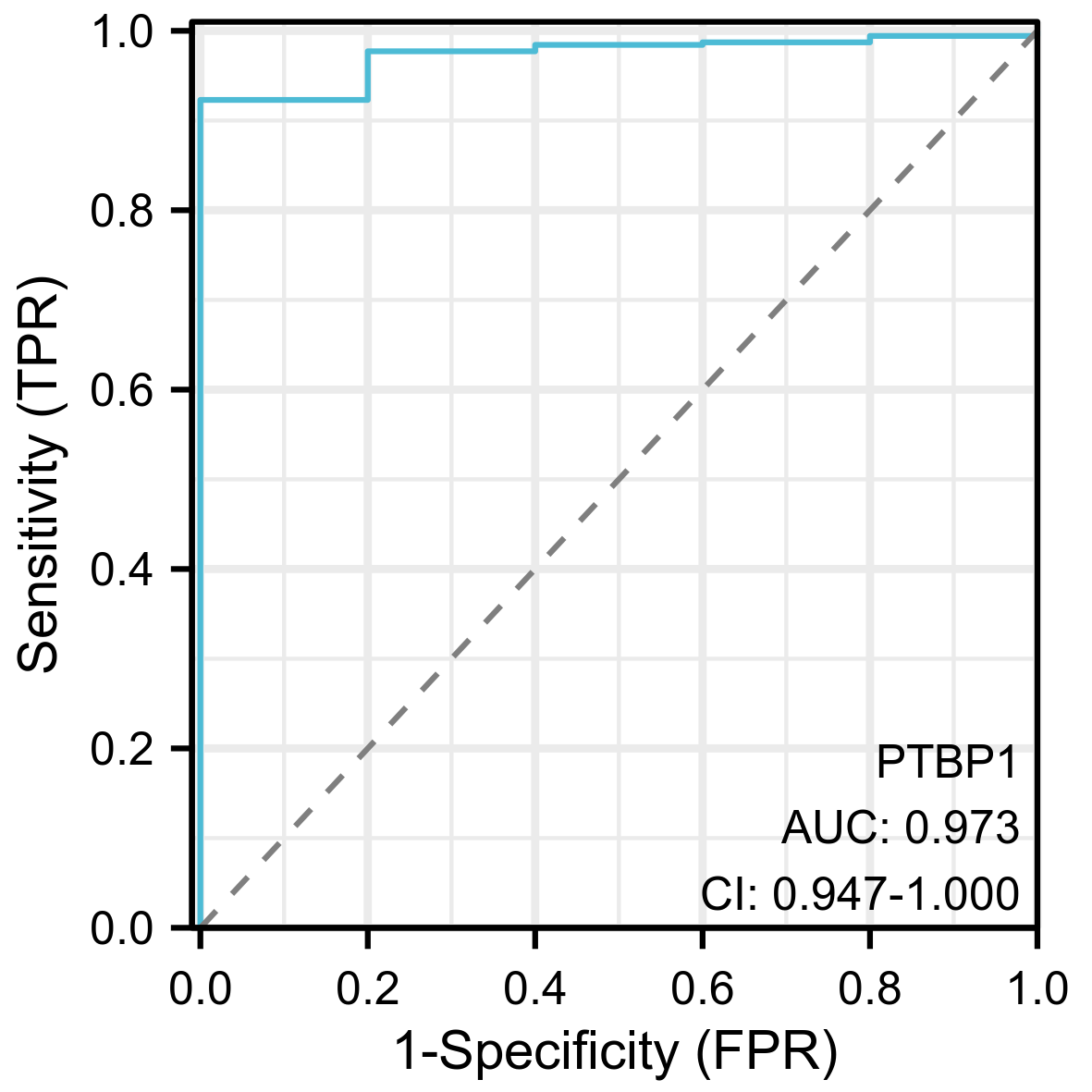

Supplement: Supplementary file 5 [file Image_3.TIFF]
